# Increasing Diversity in Residency Training Programs

**DOI:** 10.7759/cureus.25962

**Published:** 2022-06-15

**Authors:** Kundai Crites, Jasmine Johnson, Nicole Scott, Anthony Shanks

**Affiliations:** 1 Obstetrics and Gynecology, Indiana University School of Medicine, Indianapolis, USA

**Keywords:** mentorship program, residency application, racial diversity, residency recruitment, residency program, diversity and inclusion, diversity

## Abstract

Improving diversity in the healthcare workforce holds promise in improving the health outcomes of our diverse patient population. Attracting, recruiting, and retaining physicians from races and ethnicities that are historically underrepresented in medicine are vital in this effort. Increasing diversity at the graduate medical education level has the potential to positively reshape our physician personnel. In this editorial, we discuss the current state of diversity-oriented recruitment strategies for residency programs and present opportunities for future efforts.

## Editorial

Introduction

Healthcare disparities are differences in access to medical care and variations in rates of disease between population groups defined by sociodemographic characteristics [1]. It remains a national issue with historically minoritized communities, such as Black and Hispanic populations, lagging behind White populations in health outcome measures [2,3]. While equity in healthcare requires a multifaceted approach to combat issues of inequality, diversity in the healthcare workforce is one area that has the potential to lead to improvements in the care gap [4]. 

Diversity in the healthcare workforce has the potential to increase cultural competency, improve access for the underserved, and broaden research agendas [5]. Physicians from races and ethnicities that are historically underrepresented in medicine compared to the larger population include African American, Hispanic, American Indian, and certain Asian subgroups [6]. These groups are referred to as underrepresented minorities (URMs), and although they comprise one-third of the US population [7], they represent less than 15% of the residency workforce [8]. Furthermore, when examining the percentage of all practicing physicians in 2018, Hispanic, Black, and American Indian/Alaskan Native physicians comprise 5.8%, 5.0%, and 0.3% of the physician workforce, respectively [9]. It is imperative that we evaluate ways to recruit and retain physicians from racially diverse groups to improve the diversity of practicing physicians. 

As residency training programs serve as the gatekeeper for our physician workforce, efforts at improving diversity at the residency level will directly impact the diversity of practicing physicians. National organizations understand this and stress its importance [10,11]. Recently, the Accreditation Council for Graduate Medical Education has enacted several common program requirements to address issues of diversity, equity, and inclusion [12]. One requirement is that programs must implement policies and procedures that relate to recruiting and retaining a diverse and inclusive workforce of residents and faculty [13]. However, it is up to residency programs to determine their program-specific strategies. We combined a PubMed search using the search terms “diversity,” “residency,” and “recruitment” in addition to our institutional knowledge to develop this opinion piece. We plan to describe the current state of diversity-oriented recruitment efforts in residency programs and opportunities for improvement. 

What efforts have been successful? 

Focused Recruiting and Strategic Visibility on Interview Day 

Institutional buy-in is imperative for the success of diversity and equity initiatives. Once the commitment has been solidified, residency programs can then take specific steps with focused recruiting being at the forefront. An improved screening process, ensuring URM candidates meet current URM residents and continued mentoring, has proven successful for some residency programs to improve the diversity of their constituency. 

The Cardiology fellowship at the Ohio State University implemented several initiatives to improve diversity recruitment [14]. They first developed a subcommittee that consisted of program leadership in addition to a URM fellowship committee member. This group provided a holistic review for URM applicants, and following the interviews, they remained in active communication with URM candidates. They pledged to continue the mentorship of URM candidates with URM faculty during their training. Since the change in their recruitment process, they have matched at least one URM fellow per year. Before these changes, Ohio State University had never trained a URM cardiologist. 

Another example of positive changes is the Children’s Mercy Program in Kansas City. They identified that they lacked minority faculty representation on their resident selection committee and URM medical students were unlikely to meet a URM trainee or faculty member on their interview day [15]. With URM faculty input, residency leadership ensured that URM faculty were on the selection committee and, whenever possible, made a focused effort for URM candidates to meet URM faculty during their interview day. These efforts have led to a significant increase in the number of URM residents in their training program [15]. Ensuring URM faculty and current residents have a voice in the residency selection process is essential.

Implicit Bias Training for Programs and Program Leadership

Identifying implicit bias on an individual level is imperative to achieve diversity and combat racism. Graduate Medical Education (GME) training for URM candidates is particularly demanding as they are frequently the targets of microaggressions and bias [16]. Blanchard et al. propose specific strategies for GME which include incorporating inclusive pedagogy and structural competency into education and building and supporting a diverse learning environment [17]. The term “structural competency” is relatively new and encompasses recognizing the structures that shape clinical interactions and imagining structural interventions to improve outcomes [18]. Other department interventions include training new faculty members on dealing with hypervisibility, stereotype threat, and institutional racism [19]. MedEdPortal provides some excellent training modules to facilitate these discussions at a program level [20,21]. 

The implementation of implicit bias training - in addition to changes in the recruiting process - can lead to impartments in residency diversity [22]. The Nationwide Children’s Hospital Pediatric Residency Program demonstrated this when they underwent a recruitment overhaul to increase URM recruitment. This included implicit bias training for program leadership [23]. Similar to previous programs highlighted, visibility and interaction between URM applicants and residents and faculty were enhanced throughout the application cycle. Prior to the implementation of the focused changes, URM residents comprised 5% of the residency program. After the changes and increased focus on diversity recruitment, that percentage increased to 16-26% from 2018 to 2021. 

Mentorship and Retention of URM Residents

Common themes for programs that have increased the number of URM residents is early engagement and ongoing mentorship [24]. Attendance at URM fairs and conferences, such as the American Medical Education Conference, provides excellent opportunities to first engage potential applicants. Financial assistance may be of benefit to assist with travel for in-person interviews for some candidates. In addition to making initial contact and assisting in getting URM candidates to interview, these touchpoints for programs can emphasize to applicants the recognition of the importance of diversity within their program and highlight it during the interview process. It also serves to create opportunities for further mentorship and connection. 

Mentors should be approachable, honest, and available. A study of women in surgery that included URM students, residents, and faculty stressed the importance of early mentorship to facilitate increased diversity in their field [25]. Shared gender and racial identity were also appreciated by URM mentees. Programs should be cognizant of intersectionality - the overlapping of identities and the experiences of oppression and discrimination - and consider this when developing initiatives [26]. Similar gender and racial compositions of mentors and mentees can provide unique levels of support in mentorship programs. 

Once applicants have transitioned to residents in training programs, institutions can prioritize ongoing support for URM trainees and faculty. The iDREAM (Incentivizing Diverse Recruitment for Equity in Academic Medicine) Program at the Indiana University School of Medicine is one example [27]. The iDREAM programs provide financial assistance and mentorship for URM residents and attending physicians to support their developing careers in academic medicine. Mentorship is not just pairing URM trainees with URM faculty but creating meaningful connections for extra support toward career advancement. Programs should establish a culture of inclusivity and continually evaluate themselves for biases [28]. 

What should we do next?

Holistic Application Review

It is imperative that all candidates receive a holistic review of their application as opposed to solely relying on a single metric such as United States Medical Licensing Exam (USMLE) performance as an interview screen [29]. The history of standardized scores as a metric to predict academic success for URM candidates is problematic [30]. Additionally, clerkship grades - an important part of the residency application - have the potential to demonstrate racial and ethnic disparities rooted in bias [31]. The University of Washington performed an analysis of Medical Student Performance Evaluation summary words and found that after accounting for all available confounding variables, grading disparities favored White students [32]. Being mindful of the biases involved in the evaluation process is important for URM candidates to receive fair consideration for residency positions [33]. 

The Association of American Medical Colleges provides a framework on how to provide a holistic review of residency applications [29]. The review should be flexible and, importantly, individualized to assess an applicant’s abilities. Candidates should be assessed on their experiences, attributes, competencies, and scholarly metrics to see if the candidate is an individual who would contribute to a program’s mission. This method acknowledges that diversity is a core component of institutional success.

Using a holistic review of applications has been shown to increase the exposure and recruitment of URM into training sites. One Emergency Medicine program shared its experience after adjusting its application screening rubric to balance experiences across attributes [34]. Examples of attributes included the applicant’s race, ethnicity, gender identity, and socioeconomic status in addition to professionalism, motivation, and teamwork. They found that following the change to their review process, they saw increases in the number of URM applicants invited to interview (+11%), interviewed (+7.9%), and ultimately represented at the top of their rank list. Residency programs should be encouraged to review a candidate’s breadth of work, qualifications, and commitment to the specialty. Relying solely on criteria such as the USMLE and AOA status - two metrics that historically have had differences between URM and non-URM individuals - jeopardizes our ability to increase diversity in the GME workforce [35].

Faculty Development and Creating a Culture of Allyship

Training institutions must teach and expect faculty, staff, and trainees to practice antiracism [36]. There have been several successful initiatives launched at the departmental level in recent years. The Indiana University OBGYN program utilized a six-session seminar series to increase the understanding of structural racism and racial inequality [37]. Entitled AWARE (Allies Welcomed to Advance Racial Equity), it specifically called upon White faculty members to tackle the topics of structural racism, Whiteness, and anti-racist action. The sessions allowed the participants to reflect on their healthcare environment and how they could be proactive in enacting systemic change. The sessions were highly favored with 96% of attendees stating they would recommend the program to a colleague. Similarly, the OBGYN department at the University of Michigan implemented Diversity, Equity, Inclusion, and Justice (DEIJ) grand rounds along with a number of changes for ongoing faculty development [38]. Topics for the grand round talks included unconscious bias, human rights, and the importance of diversity. Feedback from these sessions was that they provided a forum for everyone in the department to have a voice and led to improvements in the overall workplace environment. 

Integrating allyship training into GME training holds promise in promoting safe spaces where diversity can flourish. Nicole Asong Nfonoyim-Hara defines allyship as “when a person of privilege works in solidarity and partnership with a marginalized group of people to help take down the systems that challenge that group’s basic rights, equal access, and ability to thrive in our society [39].” The Albany Medical Center in New York developed an Allyship in Residency Workshop [21] which was aimed at helping residents understand the definition of allyship and apply it to colleagues and across their community. An analysis of their attendees noted an increase in knowledge and comprehension of allyship competencies. 

Leveraging Social Media as a Recruiting Tool

Social media platforms such as Facebook, Instagram, and Twitter have allowed people of different backgrounds to connect and exchange information [40]. It also allows programs to showcase their commitment to DEIJ directly to prospective URM candidates. National organizations have encouraged and supported social media usage by promoting themed weeks. For example, the American College of Obstetricians and Gynecologists dedicates a week to wellness and encourage residency programs to share photos, videos, and tips on how programs maintain social wellness and work to reduce intolerance, discrimination, marginalization, and injustices [41]. As potential candidates may rely on social media for program information, residency leadership would be wise to share their vision and highlight their commitment to DEIJ at their institutions. 

Discussion

Increasing diversity in our workforce requires strategic and deliberate efforts. Most importantly, diversity recruitment is not solely about increasing the absolute number of URM residents but providing meaningful long-term support. This includes providing an inclusive environment that enriches training experiences for all and improves patient outcomes. Additionally, when making program changes, it is important to note that the burden of responsibility is disproportionally placed on minority faculty - known as the “minority tax” - which is a major source of inequity in academic medicine [42]. Taking on the responsibility of increasing diversity can hinder advancement for URM residents [23]. They may feel pressured to take on DEI efforts while sacrificing other professional endeavors. Department Chairs should ensure that URM residents and faculty have enough time and support free from clinical duties when tasked with advancing DEIJ efforts [23].

Efforts to increase diversity must be ingrained in the culture of the department and have buy-in from non-URM residents and faculty. When DEI efforts are made by non-URM individuals it not only provides support for their URM colleagues, but it exemplifies a program’s commitment to diversity, equity, and inclusion. Programs and institutions should support physicians who want to engage in antiracism work and consider hiring external experts to strengthen the cause, which would further decrease the burden on physicians of the color [36]. Departments can and should recognize diversity efforts by faculty when considering academic promotions.

Diversity in the healthcare workforce is imperative for us to reach equity in healthcare outcomes. Residency programs are one of the major pipelines for enhancing workforce diversity and they should look inward to identify modifiable factors to improve the recruitment and retention of URM candidates. A commitment to this work in recruiting practice and mentoring programs at the resident level can assist in making this a reality.

## References

[1] (2022). Agency for Healthcare Research and Quality: Disparities. https://www.ahrq.gov/topics/disparities.html.

[2] (2022). ​​​​​​​2021 National Healthcare Quality and Disparities Report. https://www.ahrq.gov/research/findings/nhqrdr/nhqdr21/index.html.

[3] Moaddab A, Dildy GA, Brown HL, Bateni ZH, Belfort MA, Sangi-Haghpeykar H, Clark SL (2018). Health care disparity and pregnancy-related mortality in the United States, 2005-2014. Obstet Gynecol.

[4] Gonzaga AM, Appiah-Pippim J, Onumah CM, Yialamas MA (2020). A framework for inclusive graduate medical education recruitment strategies: meeting the ACGME standard for a diverse and inclusive workforce. Acad Med.

[5] Cohen JJ, Gabriel BA, Terrell C (2002). The case for diversity in the health care workforce. Health Aff (Millwood).

[6] (2022). AAMC: Diversity in Medical Education. https://www.aamcdiversityfactsandfigures2016.org/./.

[7] Clayborne EP, Martin DR, Goett RR, Chandrasekaran EB, McGreevy J (2021). Diversity pipelines: the rationale to recruit and support minority physicians. J Am Coll Emerg Physicians Open.

[8] (2022). AAMC: Table B5. Number of Active MD Residents, by Race/Ethnicity (Alone or In Combination) and GME Specialty. https://www.aamc.org/data-reports/students-residents/interactive-data/report-residents/2021/table-b5-md-residents-race-ethnicity-and-specialty.

[9] Figure 18 (2022). AAMC: Diversity in Medicine: Facts and Figures 2019. https://www.aamc.org/data-reports/workforce/interactive-data/figure-18-percentage-all-active-physicians-race/ethnicity-2018.

[10] (2022). ACGME: Equity Matters. https://www.acgme.org/what-we-do/diversity-equity-and-inclusion/ACGME-Equity-Matters/.

[11] (2022). ACOG: In Solidarity: A Message to the ACOG Community. https://www.acog.org/en/news/news-articles/2020/06/in-solidarity-a-message-to-the-acog-community.

[12] (2022). ACGME: Diversity, Equity, and Inclusion. https://www.acgme.org/what-we-do/diversity-equity-and-inclusion/.

[13] (2022). ACGME: Common Program Requirements. https://www.acgme.org/globalassets/PFAssets/ProgramRequirements/CPRResidency2021.pdf.

[14] Auseon AJ, Kolibash AJ Jr, Capers Q (2013). Successful efforts to increase diversity in a cardiology fellowship training program. J Grad Med Educ.

[15] Lewis T, Tolbert J, Jones BL (2020). Increasing resident racial and ethnic diversity through targeted recruitment efforts. J Pediatr.

[16] Osseo-Asare A, Balasuriya L, Huot SJ (2018). Minority resident physicians' views on the role of race/ethnicity in their training experiences in the workplace. JAMA Netw Open.

[17] Blanchard AK, Blanchard JC, Suah A, Dade A, Burnett A, McDade W (2022). Reflect and reset: Black academic voices call the graduate medical education community to action. Acad Med.

[18] Metzl JM, Hansen H (2014). Structural competency: theorizing a new medical engagement with stigma and inequality. Soc Sci Med.

[19] Doll KM, Thomas CR Jr (2020). Structural solutions for the rarest of the rare - underrepresented-minority faculty in medical subspecialties. N Engl J Med.

[20] Gonzalez CM, Walker SA, Rodriguez N, Noah YS, Marantz PR (2021). Implicit bias recognition and management in interpersonal encounters and the learning environment: a skills-based curriculum for medical students. MedEdPORTAL.

[21] Martinez S, Araj J, Reid S (2021). Allyship in residency: an introductory module on medical allyship for graduate medical trainees. MedEdPORTAL.

[22] Akhiyat S, Cardwell L, Sokumbi O (2020). Why dermatology is the second least diverse specialty in medicine: How did we get here?. Clin Dermatol.

[23] Hoff ML, Liao NN, Mosquera CA (2022). An initiative to increase residency program diversity. Pediatrics.

[24] Escalante E, Smiley Y, Agrawal D, Teach SJ, Cora-Bramble D, Barber A (2021). Increasing pediatric residency class diversity to improve patient outcomes and address structural racism. Acad Med.

[25] Mahendran GN, Walker ER, Bennett M, Chen AY (2022). Qualitative study of mentorship for women and minorities in surgery. J Am Coll Surg.

[26] (2022). University of Bristol: Equality, Diversity, Inclusion: Intersectionality. https://www.bristol.ac.uk/inclusion/intersectionality/.

[27] (2022). Indiana University School of Medicine: iDREAM | Diversity. https://medicine.iu.edu/about/diversity/programs/recruitment-retention/idream.

[28] Davenport D, Alvarez A, Natesan S (2022). Faculty recruitment, retention, and representation in leadership: an evidence-based guide to best practices for diversity, equity, and inclusion from the Council of Residency Directors in Emergency Medicine. West J Emerg Med.

[29] (2022). AAMC: Holistic Review. https://www.aamc.org/services/member-capacity-building/holistic-review.

[30] Tough P (2022). Tough P. The Inequality Machine. https://www.paultough.com/books/the-inequality-machine/.

[31] (2022). Gaslighting of Black Medical Trainees Makes Residency Something to ‘Survive’. https://www.statnews.com/2022/03/10/gaslighting-black-medical-trainees-residency/.

[32] Low D, Pollack SW, Liao ZC, Maestas R, Kirven LE, Eacker AM, Morales LS (2019). Racial/ethnic disparities in clinical grading in medical school. Teach Learn Med.

[33] Tidwell J, Yudien M, Rutledge H, Terhune KP, LaFemina J, Aarons CB (2022). Reshaping residency recruitment: achieving alignment between applicants and programs in surgery. J Surg Educ.

[34] Sungar WG, Angerhofer C, McCormick T, Zimmer S, Druck J, Kaplan B, Ward-Gaines J (2021). Implementation of holistic review into emergency medicine residency application screening to improve recruitment of underrepresented in medicine applicants. AEM Educ Train.

[35] Williams M, Kim EJ, Pappas K, Uwemedimo O, Marrast L, Pekmezaris R, Martinez J (2020). The impact of United States Medical Licensing Exam (USMLE) step 1 cutoff scores on recruitment of underrepresented minorities in medicine: a retrospective cross-sectional study. Health Sci Rep.

[36] Argueza BR, Saenz SR, McBride D (2021). From diversity and inclusion to antiracism in medical training institutions. Acad Med.

[37] Tucker Edmonds B, Neal C, Shanks A (2021). Allies Welcomed to Advance Racial Equity (AWARE) faculty seminar series: program design and implementation. J Med Educ Curric Dev.

[38] Mmeje O, Price EA, Johnson TR, Fenner DE (2020). Galvanizing for the future: a bottom-up departmental approach to diversity, equity, and inclusion. Am J Obstet Gynecol.

[39] (2022). NIH: What Is Allyship? | Office of Equity, Diversity and Inclusion. https://www.edi.nih.gov/blog/communities/what-allyship.

[40] October 3 BK, Pm 2019 at 6: 50 (2022). Strategic Finance: Social Media as a Means to Diversity. https://sfmagazine.com/post-entry/october-2019-social-media-as-a-means-to-diversity/.

[41] (2022). ACOG National Wellness Week. https://www.acog.org/en/education-and-events/creog/national-wellness-week.

[42] Rodríguez JE, Campbell KM, Pololi LH (2015). Addressing disparities in academic medicine: what of the minority tax?. BMC Med Educ.

